# Neurologic Complications in Adult Post-cardiotomy Cardiogenic Shock Patients Receiving Venoarterial Extracorporeal Membrane Oxygenation: A Cohort Study

**DOI:** 10.3389/fmed.2021.721774

**Published:** 2021-08-11

**Authors:** Dengbang Hou, Hong Wang, Feng Yang, Xiaotong Hou

**Affiliations:** Center for Cardiac Intensive Care, Beijing Institute of Heart, Lung and Blood Vessel Diseases, Beijing Anzhen Hospital, Capital Medical University, Beijing, China

**Keywords:** extracorporeal membrane oxygenation, post-cardiotomy cardiogenic shock, neurological complication, lowest systolic blood pressure, in-hospital mortality

## Abstract

**Background:** This study aims to describe the prevalence of neurologic complications and hospital outcome in adult post-cardiotomy cardiogenic shock (PCS) patients receiving veno-arterial extracorporeal membrane oxygenation (V-A ECMO) support and factors associated with such adverse events.

**Methods:** Four hundred and fifteen adult patients underwent cardiac surgery and received V-A ECMO for more than 24 h because of PCS. Patients were divided into two groups: those who developed a neurological complication and those who did not (control group). Multivariable logistic regression was performed to identify factors independently associated with neurologic complications.

**Results:** Neurologic complications occurred in 87 patients (21.0%), including cerebral infarction in 33 patients (8.0%), brain death in 30 patients (7.2%), seizures in 14 patients (3.4%), and intracranial hemorrhage in 11 (2.7%) patients. In-hospital mortality in patients with neurologic complications was 90.8%, compared to 52.1% in control patients (*p* < 0.001). In a multivariable model, the lowest systolic blood pressure (SBP) level pre-ECMO (OR, 0.89; 95% CI: 0.86–0.93) and aortic surgery combined with coronary artery bypass grafting (OR, 9.22; 95% CI: 2.10–40.55) were associated with overall neurologic complications. Age (OR, 1.06; 95% CI: 1.01–1.12) and lowest SBP (OR, 0.81; 95% CI: 0.76–0.87) were correlative factors of brain death. Coagulation disorders (OR, 9.75; 95% CI: 1.83–51.89) and atrial fibrillation (OR, 12.19; 95% CI: 1.22–121.61) were shown to be associated independently with intracranial hemorrhage, whereas atrial fibrillation (OR, 8.15; 95% CI: 1.31–50.62) was also associated with cerebral infarction.

**Conclusions:** Neurologic complications in adult PCS patients undergoing V-A ECMO support are frequent and associated with higher in-hospital mortality. Identified risk factors of neurologic complications might help to improve ECMO management and might reduce their occurrence.

## Introduction

Veno-arterial extracorporeal membrane oxygenation (V-A ECMO) is an effective technique to rescue patients with refractory cardiogenic shock or cardiac arrest (1–4). Despite the significantly increasing use and experience in recent years, V-A ECMO is still associated with very high in-hospital mortality (40–60%) and high rate of complications. Of these, bleeding, renal failure, infection, and neurologic complications, often result in poor outcomes or permanent disability (5–8). Previous studies have shown that the mortality in V-A ECMO patients associated with neurologic complications was high (9–13). However, the patients enrolled in these studies were from the Extracorporeal Life Support Organization (ELSO) registry or the complication profiles of detailed V-A ECMO indications were well not well-defined or including various V-A ECMO settings (9–13). One of the most common V-A ECMO indications is post-cardiotomy cardiogenic shock (PCS). Better understanding of the neurologic complications in PCS adult patients receiving V-A ECMO support might be meaningful to elucidate this peculiar aspect and improve the ECMO management in this challenging setting.

This study, therefore, aimed to assess the prevalence of cerebral injury and its influence on outcomes in adult PCS patients undergoing V-A ECMO support. Furthermore, independent risk factors of neurologic complications were also investigated.

## Materials and Methods

### ECMO Setting and Patient Profile

**Figure 1:**
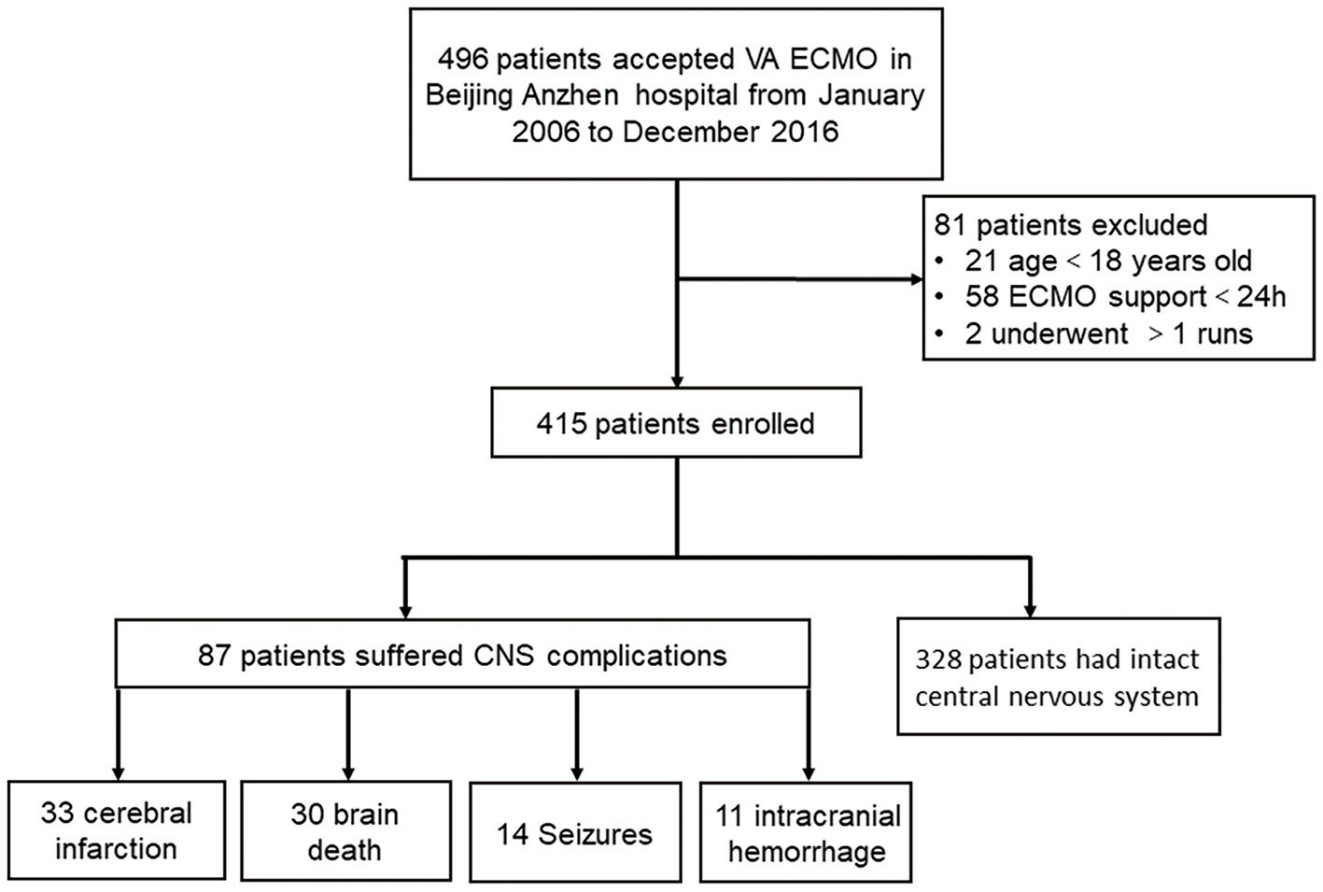
Study flow. CNS, central nervous system.

The present study was a retrospective cohort study conducted at Beijing Anzhen Hospital, Capital Medical University. Forty-two thousand six hundred and sixty-eight adult patients (>18 years old) received cardiac surgery in our center from January 2006 to December 2016. Four hundred and ninety-six underwent V-A ECMO because of PCS. Of those, 21 patients aged 17 years or younger were excluded. Fifty-eight patients undergoing ECMO for <24 h were excluded because of the lack of complete central nervous system (CNS) assessment. Two patients undergoing more than one ECMO run were also excluded to avoid bias from confounders contributing to the severity of illness. Finally, 415 adult patients requiring V-A ECMO were included in this study. Patients were categorized according to the in-hospital occurrence (the neurological complication group) or absence of neurologic complications (the control group), and the two groups were compared (Figure 1). Data were extracted from the prospective institutional registry database of ECMO patients. This study was approved by Beijing Anzhen Hospital human research ethics committee (Ethics number: 2016018X). Because this was a retrospective observational study, the individual patients' consent was waived.

Post-cardiotomy cardiogenic shock patients included: (1) those who could not be weaned from cardiopulmonary bypass (CPB); (2) those presenting low cardiac output syndrome (LCOS) after CPB, cardiac arrest, or arrhythmias and hemodynamic instability despite satisfactory cardiac surgical procedure and conventional anti-arrhythmia therapy in the operating room; (3) those with delayed LCOS or cardiac arrest in intensive care unit (ICU) (14). Postoperative LCOS was defined as a systolic blood pressure (SBP) <90 mmHg for at least 30 min with a severe reduction in cardiac index (<1.8 L/min/m^2^) and elevated left or right ventricular filling pressures, or inadequate peripheral organ perfusion (pH < 7.3, serum lactate > 2 mmol/L, cool extremities, urine output <30 mL/h, and altered mental status), acute pulmonary congestion or edema despite adequate/appropriate fluid administration and pharmacologic agents or intra-aortic balloon pump (IABP) (14).

### V-A ECMO Implantation Techniques

The decision to use V-A ECMO was made by the cardiac surgeon and ECMO team. V-A ECMO was setup by the experienced ECMO team members. The femoral vessels (vein and artery) were cannulated with Fr 17–21 draining cannulae, and Fr 15–19 perfusion cannulae (Medtronic, Minneapolis, MN) by surgical cut-down. An additional 7F catheter was systematically inserted distally to the cannulated femoral artery site to perfuse the limb. Intrathoracic cannulation strategy has not been used in the 415 patients.

### Patient Management

The detailed management of patients under V-A ECMO was previously described (14, 15). Heparin was used for anticoagulation. A heparin bolus (5,000 IU) was injected before cannulation. After V-A ECMO initiation, if surgical site bleeding could be controlled or thoracic drainage was <0.5 ml/kg/min, continuous intravenous infusion of unfractionated heparin was given to the patients as early as possible to maintain the activated clotting time (ACT) between 180 and 220 s, or activated partial thromboplastin time (aPTT) in the range of 60–80 s. Therapeutic hypothermia (32–35°C) was initiated during the first 24 h in case of extracorporeal cardiopulmonary resuscitation (ECPR) patients. The partial pressure of carbon dioxide (PaCO_2_) was maintained between 35 and 45 mmHg before airway extubation.

Coagulation disorders could occur at any time of the perioperative period. In this study, we analyzed the coagulation disorders, defined as platelets <20 × 10^9^/L, fibrinogen <1.5 g/L and prothrombin time <30% of the standard value (16), before and during ECMO support. The definition and/or treatment of ECMO-related complications, such as major bleeding, lower-limb ischemia or compartment syndrome requiring fasciotomy, renal failure requiring renal replacement therapy, and significant infection, were described in the previous reports (14, 17).

### Definition, Monitoring, and Treatment of Neurologic Complications

We defined neurologic complications as any clinical event occurring during the V-A ECMO support, including any clinical sign suggestive of stroke, brain death, and seizures despite sedation (11–13, 17). Routine neurological examinations were performed at least twice a day by the ICU doctors and the nurses in charge of the patients during sedation interruption or after sedation withdrawal, including response to verbal orders or pain, tendon reflexes, brainstem reflexes, eye movement, and pupil size and their light reflection. When abnormal signs were detected (such as pupil dilatation, convulsion of the limbs, delirium confusion, no awakening after sedation withdrawal, etc.), a cerebral computed tomography (CT) scan was performed within 6 h, and a neurologist was consulted immediately to perform neurocognitive test (18). The diagnosis of cerebral infarction or intracranial hemorrhage was determined by a neurologist analyzing CT scan images. Brain death, an irreversible cessation of the functions of the entire brain, including the brain stem (19) was defined according to the diagnostic criteria set by the American Academy of Neurology (AAN) (19, 20). In addition to neurologic examination, EEG and transcranial Doppler were performed to confirm the electrical activity loss or the loss of cerebral blood flow. Seizures were identified by a neurologist by means of at least 30 min continuous EEG monitoring and clinical features. When a neurological lesion was diagnosed, it would be treated according to the neurologist's consultation.

### ECMO Weaning

Weaning from V-A ECMO support was based on the clinical and laboratory evidences of recovery of cardiac and pulmonary function, including that pulsatile arterial waveform was maintained (pulse pressure >20 mmHg) for over 24 h, and LV ejection fraction was >20–25% or not worsened right heart function and no significant arterial blood O_2_ saturation when the ECMO flow was reduced to <1.5 L/min according to conventional protocol (15). Cardiac function and blood gas were continuously monitored during the weaning process. V-A ECMO was removed, and the femoral artery was primarily repaired in the operating room. Weaning off ECMO was considered successful when a patient survived V-A ECMO explantation for at least 48 h (15).

### Statistical Analysis

Categorical data are reported as numbers and percentages. Continuous variables are expressed as mean ± standard deviations for normally distributed variables, or as median and interquartile range (IQR) for non-normally distributed variables. Normality of distribution was assessed by the Kolmogorov–Smirnov test. Categorical variables were compared with chi-square or Fisher's exact tests. Continuous variables were compared with two-tailed Student's *t*-test or Mann–Whitney *U*-test. Univariable and stepwise multivariate logistic regression analyses of baseline characteristics, pre-ECMO, and ECMO-related risk factors for neurologic complications were performed by calculating the odds ratio (OR) with 95% confidence interval (CI). Variables with *p* < 0.05 during univariable analysis were entered in multivariate logistic regression. Variables were retained in the model if the adjusted *p*-value was < 0.05. The maximum value of Youden's index was used to determine the threshold of lowest SBP before VA ECMO initiation. Analyses were performed using IBM SPSS Statistics v22.0 software (IBM Corp, Chicago, IL). *p* < 0.05 defined statistical significance.

## Results

Two hundred and sixty-eight patients (64.6%) were successfully weaned off V-A ECMO, and 165 patients (39.8%) survived to discharge. Eighty-seven patients (21.0%) suffered from neurologic complications during V-A ECMO support (Figure 1). Thirty-three patients had preexisting neurological comorbidities (32 had a history of cerebral infarction, and 1 had suffered intracranial hemorrhage).

### Baseline Characteristics

In comparing the baseline characteristics between the neurological complication group and the control group (Table 1), we found significant differences in age and body mass index (BMI). Patients experiencing neurological complications were older and had a higher ratio of BMI ≥ 30 (*p* < 0.05). The rates of hypertension and peripheral arterial disease were also higher in the neurological complication group (*p* < 0.01). Patients with atrial fibrillation appeared more frequently in the neurological complication group (*p* < 0.05). Patients in the neurological complication group had a higher ratio of cardiopulmonary resuscitation (CPR) before V-A ECMO initiation (43.7 vs. 20.7%, *p* < 0.001), and the lowest SBP before V-A ECMO start was lower in the neurological complication group [70.0 (65.0, 80.0) vs. 80.0 (75.0, 80.0) mmHg, *p* < 0.001]. Receiving aortic surgery combined with coronary artery bypass grafting (CABG) was more common in the neurological complication group (6.9 vs. 1.2%, *p* < 0.01). In our study, no heart transplantation recipients developed neurologic complications (*p* < 0.05; Table 1).

**Table 1:** Demographics, baseline characteristics, and pre-extracorporeal membrane oxygenation information.

**Variable**	**Neurological complication group (*n* = 87)**	**Control group (*n* = 328)**	***p*** **-value**
**Demographics and baseline characteristics**			
Age (years)	61.0 [51.0, 66.0]	55.0 [45.3, 63.8]	0.002
Older age (≥65 year)	27 (31.0)	80 (24.4)	0.208
Gender (male)	56 (64.4)	225 (68.6)	0.453
Body mass index (kg/m^2^)	24.3 ± 3.7	23.7 ± 3.5	0.154
Obesity (BMI ≥ 30)	15 (17.2)	30 (9.1)	0.031
Smoking	35 (40.2)	146 (44.5)	0.460
**Comorbidities**			
Hypertension	47 (54.0)	117 (35.7)	0.002
Hyperlipidemia	7 (8.0)	23 (7.0)	0.746
Diabetes mellitus	20 (23.0)	63 (19.2)	0.441
Coronary artery disease	48 (55.2)	161 (49.1)	0.325
Peripheral arteria disease	21 (24.1)	36 (11.0)	0.001
COPD	2 (2.3)	4 (1.2)	0.460
Abnormal liver function	0	4 (1.2)	0.300
Atrial fibrillation	3 (3.4)	2 (0.6)	0.031
Preexisting neurological comorbidities	8 (9.2)	25 (7.6)	0.630
**Pre-ECMO situation**			
Intra-aortic balloon pump	49 (56.3)	187 (57.0)	0.908
CPR history before ECMO	38 (43.7)	68 (20.7)	<0.001
Lowest SBP (mmHg)	70.0 [65.0, 80.0]	80.0 [75.0, 80.0]	<0.001
Blood glucose (mg/dl)	256.0 [198.0, 311.0]	243.0 [189.0, 289.0]	0.177
**Pre-ECMO cardiac procedures**			
CABG	41 (47.1)	122 (37.2)	0.092
Valve replacement	20 (23.0)	85 (25.9)	0.577
Aortic surgery	7 (8.0)	14 (4.3)	0.153
CHD repair	2 (2.3)	10 (3.0)	0.711
CABG and valve replacement	7 (8.0)	36 (11.0)	0.425
CABG and aortic surgery	6 (6.9)	4 (1.2)	0.002
CABG and CHD repair	0	1 (0.3)	0.606
Valve replacement and CHD repair	0	9 (2.7)	0.118
Valve replacement and aortic surgery	0	5 (1.5)	0.247
CABG combined valve replacement and CHD repair	0	1 (0.3)	0.606
CABG combined valve replacement and aortic surgery	1 (1.1)	2 (0.6)	0.597
CABG combined aortic surgery and CHD repair	0	1 (0.3)	0.606
Heart transplantation	0	23 (7.0)	0.011
Atrial/ventricular thrombus clearance	3 (3.4)	10 (3.0)	0.849
Pulmonary embolism	0	10 (3.0)	0.099
Re-operation	1 (1.1)	9 (2.7)	0.389

*Results are expressed as mean ± SD, number (%) or median [27th−75th percentile interquartile range]*.

*CABG, coronary artery bypass grafting; COPD, chronic obstructive pulmonary disease; CPR, cardiopulmonary resuscitation; SBP, systolic blood pressure, CHD, congenital heart disease*.

### ECMO Indications and Outcomes of Patients With and Without Neurologic Complications

The proportion of patients receiving V-A ECMO for failure to wean off CPB was significantly lower in the neurological complication group (41.4 vs. 55.2% in the control group, *p* < 0.05). In addition, there were 61 ECPR patients, the incidence of neurological complications of these patients was 39.3%. The proportion of ECPR patients was significantly higher in the neurological complication group (27.6 vs. 11.3% in the control group, *p* < 0.01; Table 2).

**Table 2:** VA-ECMO indications and outcomes.

**Variable**	**Neurological complication group (*n* = 87)**	**Control group (*n* = 328)**	***p*** **-value**
**ECMO implantation**			
Failure to wean off CPB	36 (41.4)	181 (55.2)	0.022
LCOS in ICU	27 (31.0)	110 (33.5)	0.659
ECPR	24 (27.6)	37 (11.3)	<0.001
**Transfusion**			
RBC (U)	26.0 [14.0, 35.0]	23.5 [14.0, 32.0]	0.417
FFP (ml)	2400.0 [1400.0, 3400.0]	2000.0 [1400.0, 3000.0]	0.309
**Complications**			
Renal failure need CRRT	46 (52.9)	155 (47.3)	0.351
Lower extremities ischemia	15 (17.2)	26 (7.9)	0.010
Femoral artery embolism	1 (1.1)	3 (0.9)	0.842
Cannulate site hemorrhage	4 (4.6)	28 (8.5)	0.221
Retroperitoneal hematoma	0	1 (0.3)	0.606
Major bleeding of other reasons	11 (12.6)	43 (13.1)	0.909
Surgical incision infection	3 (3.4)	28 (8.5)	0.113
Sepsis	16 (18.4)	76 (23.2)	0.327
Lower phlebothrombosis	0	1 (0.3)	0.606
Re-thoracotomy for hemostasis	37 (42.5)	136 (41.5)	0.858
Tracheostomy	41 (47.1)	126 (38.4)	0.141
**Outcomes**			
Weaning from ECMO	31 (35.6)	237 (72.3)	<0.001
Duration of ECMO (h)	97.0 [50.0, 128.0]	95.5 [65.0, 137.0]	0.388
Survival to discharge	8 (9.2)	157 (47.9)	<0.001
Duration of MV (h)	137.0 [72.0, 216.0]	121.5 [52.5, 210.0]	0.181
ICU length of stay (h)	170.0 [95.0, 245.0]	169.5 [96.0, 264.0]	0.386
Hospital stay (d)	17.0 [12.0, 25.0]	25.0 [17.0, 36.75]	<0.001

*Results are expressed as mean ± SD, number (%) or median [27th−75th percentile interquartile range]*.

*CPB, cardiopulmonary bypass; LCOS, low cardiac output syndrome; ECMO, extracorporeal membrane oxygenation; ECPR, extracorporeal cardiopulmonary resuscitation; CRRT, continuous renal replacement therapy; RBC, red blood cells, ICU, intensive care unit; FFP, fresh frozen plasma; MV, mechanical ventilation*.

**Figure 2:**
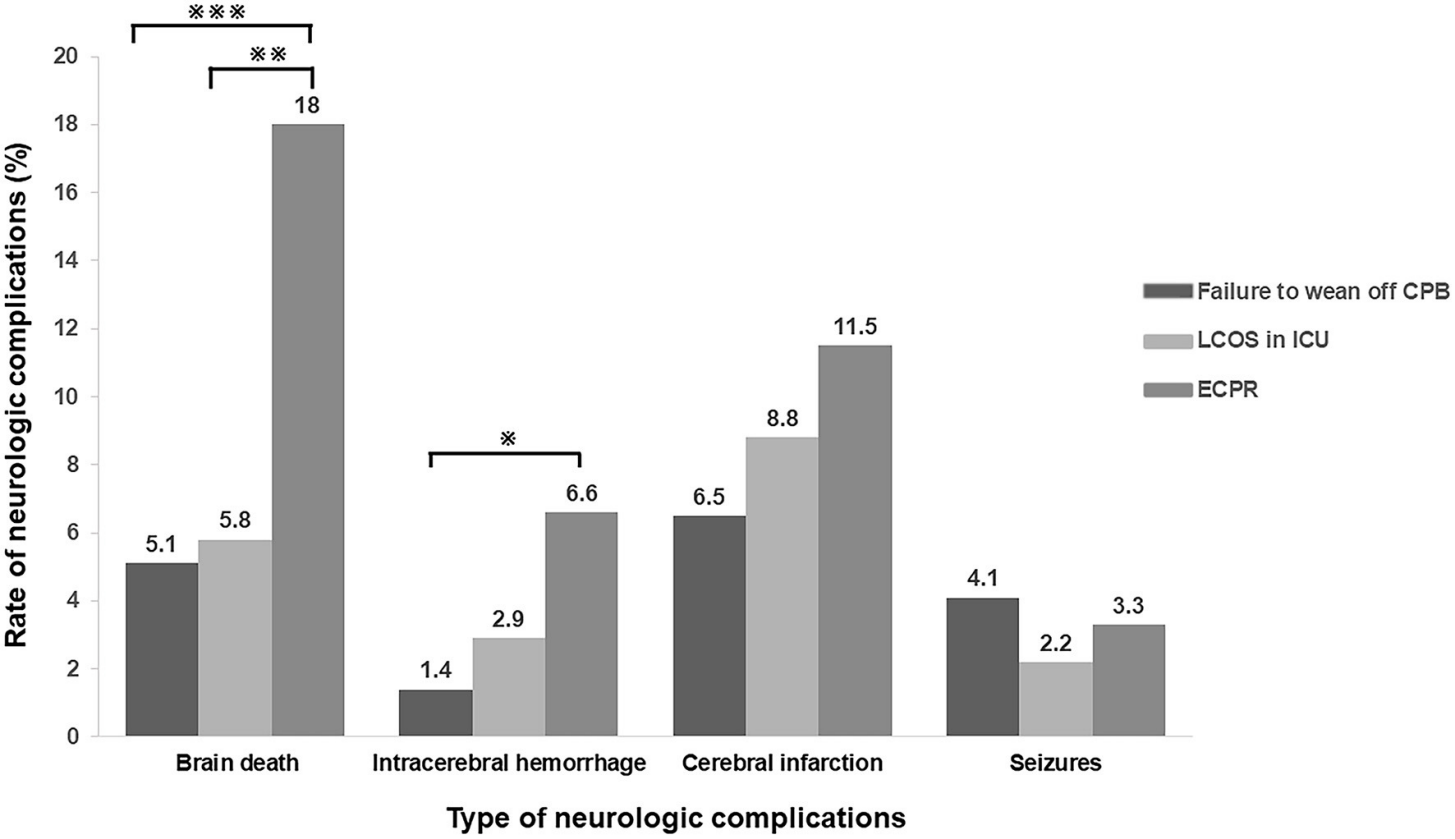
Incidence of neurologic complications in different types of ECMO indications. ECPR patients had a higher rate of brain death, intracranial hemorrhage and cerebral infarction when compared with the patients with other indications. (^※^*p* < 0.05; ^※※^*p* < 0.01; ^※※※^*P* < 0.005) CPB, cardiopulmonary bypass; LCOS, low cardiac output syndrome; ICU, intensive care unit; ECPR, extracorporeal cardiopulmonary resuscitation.

Further analysis was performed to detect the differences in the type of neurologic complications among the patients who experienced failure to wean off CPB, LCOS and ECPR. ECPR patients had a higher rate of brain death (18.0 vs. 5.1% in the patients failure to wean off CPB or 5.8% in the patients of LCOS, *p* < 0.01, respectively; Figure 2).

Blood transfusion and severe bleeding were similar between the two groups (*p* > 0.05). Lower-extremity ischemia occurred more often in the neurological complication group (17.2 vs. 7.9% in the control group, *p* < 0.05). There were no significant differences in the duration of ECMO, mechanical ventilation (MV), and ICU stay. However, the overall hospital stay in the neurological complication group was significantly shorter than that in the control group [17.0 (12.0, 25.0) days vs. 25.0 (17.0, 36.75) days, *p* < 0.001] (Table 2).

**Figure 3:**
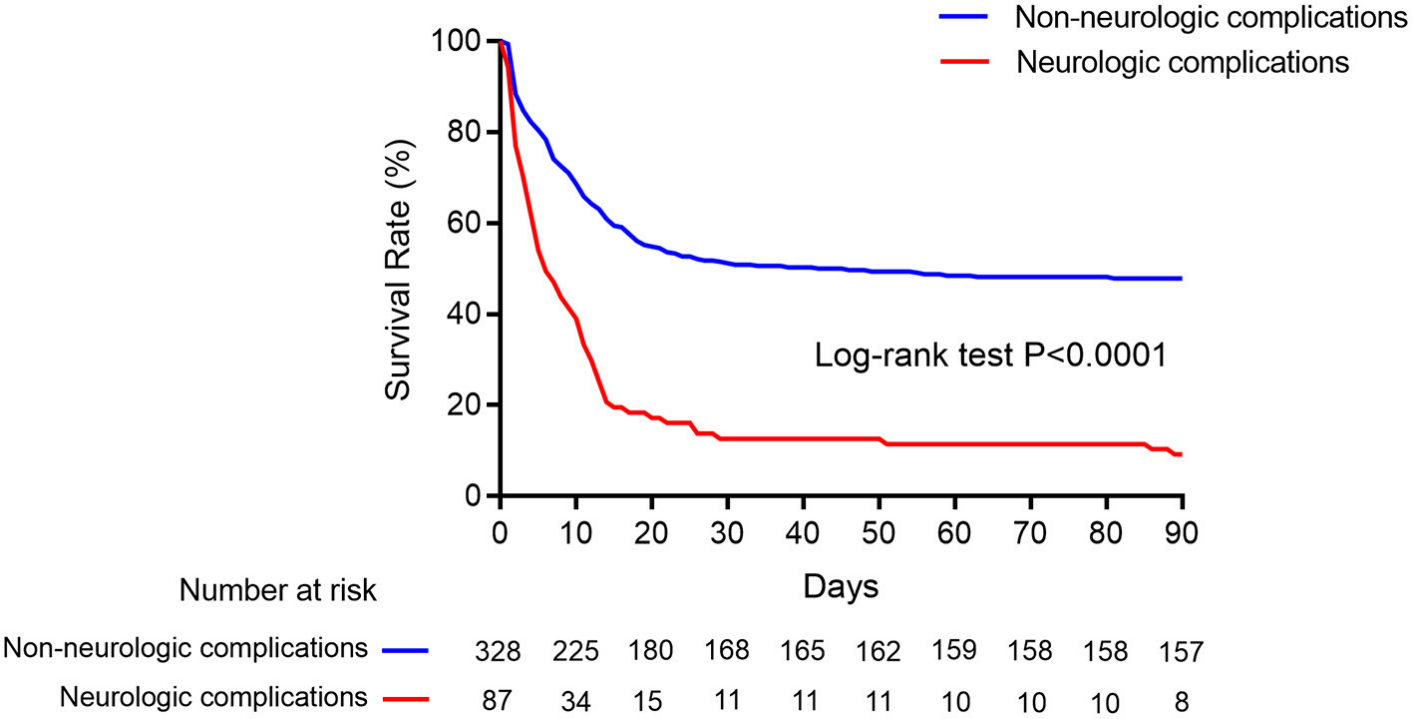
Kaplan–Meier cumulative in-hospital mortality after ECMO initiation. Kaplan–Meier survival curves showed in-hospital mortality in ECMO patients with neurologic complications (red line) and without (blue line).

The rate of successful weaning from ECMO (35.6 vs. 72.3%, *p* < 0.001) and survival to discharge (9.2 vs. 47.9%, *p* < 0.001) in the neurological complication group were significantly lower than those in the control group (Table 2; Figure 3).

### Prevalence and Prognosis of Different Type of Neurologic Complications

Among all kinds of neurologic complications, cerebral infarction was the most frequent (33 patients, 8.0%), followed by brain death (30, 7.2%), seizures (14, 3.4%), and intracranial hemorrhage (11, 2.7%), respectively. Two patients presented two kinds of neurologic complications at the same time.

**Figure 4:**
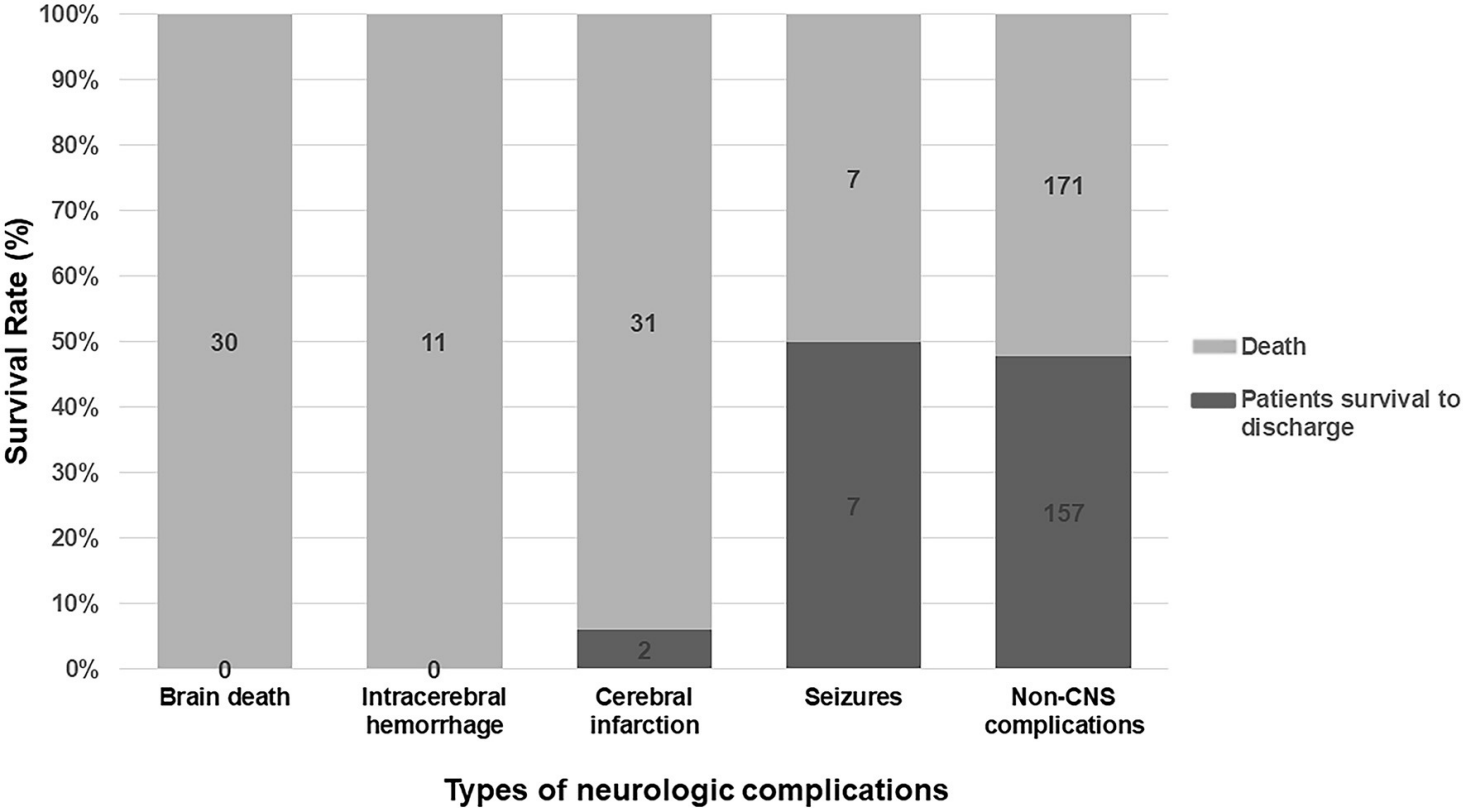
Subgroup analysis for survival rate in different kinds of neurologic complications. Patients with brain death, intracranial hemorrhage, and cerebral infarction had a catastrophic outcome. Patients suffered seizures had a similar prognosis to patients in the control group.

Poor survival was observed in the patients with brain death, intracranial hemorrhage, and cerebral infarction (*p* < 0.001 compared with the control group). Patients with seizures had similar survival rate as compared to patients in the control group (Figure 4).

### Risks Factors Associated With Neurologic Complications

Multivariate logistic regression analysis showed that the lowest SBP level before VA ECMO initiation (OR, 0.89; 95% CI: 0.86–0.93; *p* < 0.001) and aortic vascular surgery combined with CABG (OR, 9.22; 95% CI: 2.10–40.55; *p* < 0.01) were associated with neurologic complications. Although ECPR was more common in the neurological complication group, it was not a risk factor at multivariable analysis (Table 3).

**Table 3:** Univariable and multivariable analyses of factors associated with neurologic complications.

**Factor**	**Univariable analysis**		***P*** **-value**	**Multivariable analysis**		***P*** **-value**
	**OR**	**95% CI**		**OR**	**95% CI**	
**Overall neurologic complications**						
Age	1.03	1.01–1.05	0.003			
Obesity (BMI ≥ 30)	2.07	1.06–4.05	0.034			
Hypertension	2.11	1.31–3.40	0.002			
Peripheral arterial disease	2.60	1.43–4.75	0.002			
CPR history before ECMO	2.97	1.80–4.89	<0.001			
Lowest SBP	0.89	0.86–0.92	<0.001	0.89	0.86–0.93	<0.001
CABG combined with aortic surgery	6.00	1.65–21.76	0.006	9.22	2.10–40.55	0.003
ECPR	3.00	1.68–5.36	<0.001			
Failure to wean off CPB	0.57	0.36–0.93	0.023			
**Brain death**						
Age	1.06	1.02–1.11	0.002	1.06	1.01–1.12	0.022
Obesity (BMI ≥ 30)	2.78	1.12–6.90	0.028			
Peripheral arterial disease	3.14	1.35–7.30	0.008			
CPR history before ECMO	6.95	3.14–15.42	<0.001			
Lowest SBP	0.81	0.77–0.86	<0.001	0.81	0.76–0.87	<0.001
CABG	2.89	1.34–6.25	0.007			
ECPR	3.88	1.74–8.63	0.001			
**Intracranial hemorrhage**						
Coagulation disorders	8.76	1.67–45.85	0.010	9.75	1.83–51.89	0.008
Atrial fibrillation	10.00	1.02–97.71	0.048	12.19	1.22–121.61	0.033
**Cerebral infarction**						
Atrial fibrillation	8.15	1.31–50.62	0.024			
**Seizures**						
Hyperlipidemia	5.75	1.69–19.60	0.005			

*BMI, body mass index; CABG, coronary artery bypass grafting; CPB, cardiopulmonary bypass; CPR, cardiopulmonary resuscitation; ECMO, extracorporeal membrane oxygenation; ECPR, extracorporeal cardiopulmonary resuscitation; SBP, systolic blood pressure*.

**Figure 5:**
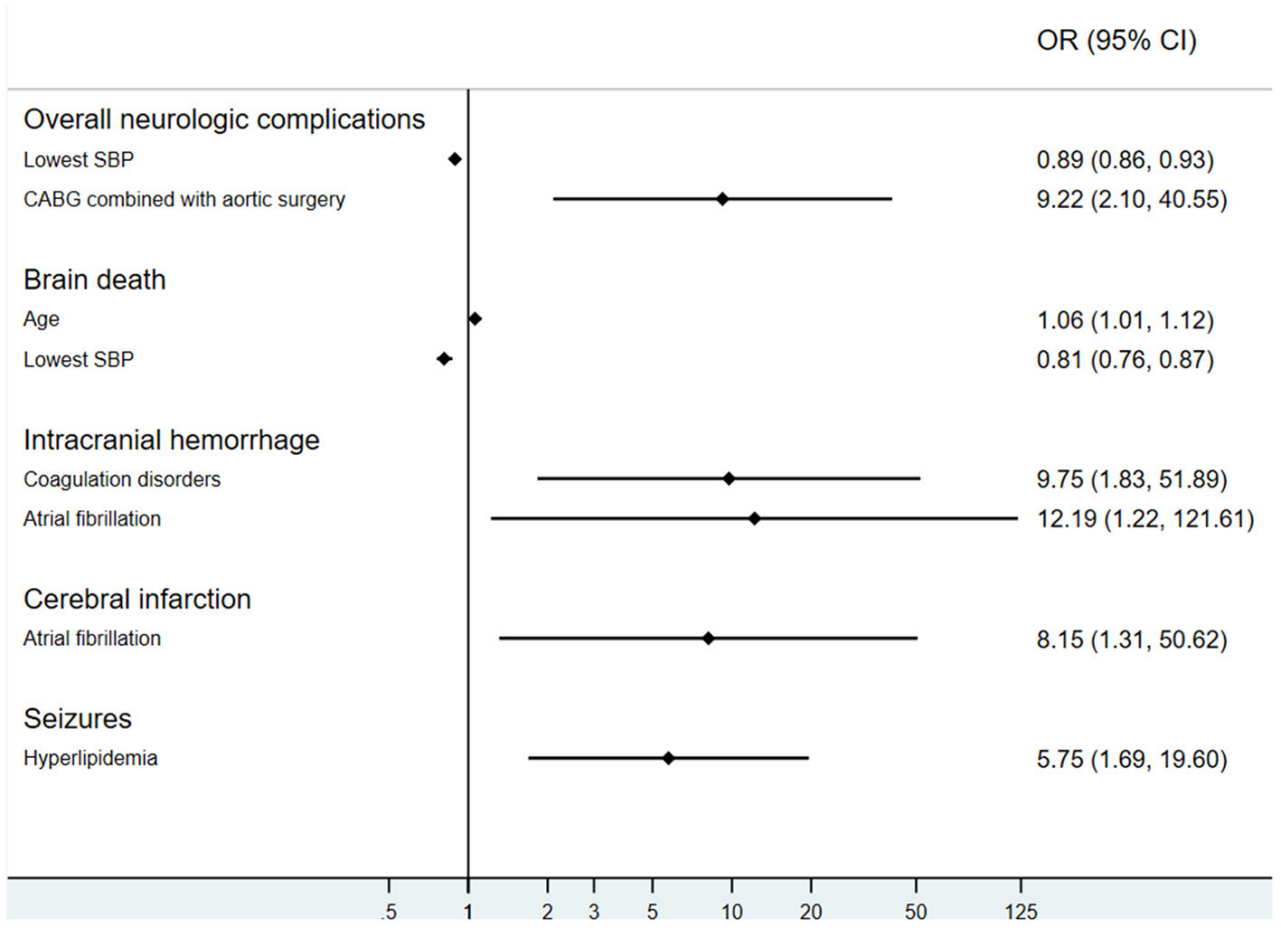
Risk factors of neurologic complications during extracorporeal membrane oxygenation. SBP, systolic blood pressure; CABG, coronary artery bypass grafting.

Age (OR, 1.06; 95% CI: 1.01–1.12, *p* < 0.05) and lowest SBP (OR, 0.81, 95% CI: 0.76–0.87, *p* < 0.001) were correlative factors of brain death. Coagulation disorders (OR, 9.75, 95% CI: 1.83–51.89, *p* < 0.01) and atrial fibrillation (OR, 12.19, 95% CI: 1.22–121.61, *p* < 0.05) could influence the incidence of intracranial hemorrhage. The occurrence of cerebral infarction might also be affected by atrial fibrillation (OR, 8.15, 95% CI: 1.31–50.62, *p* < 0.05). Hyperlipidemia patients had increased odds for seizures (OR, 5.75, 95% CI: 1.69–19.60, *p* < 0.01). It is noteworthy that preexisting neurological comorbidities were not risk factors of neurologic complications on ECMO (OR, 1.23, 95% CI: 0.53–2.83, *p* > 0.05; Figure 5).

### Threshold of the Lowest SBP Level Before V-A ECMO Initiation in the Prediction of Neurologic Complications

**Figure 6:**
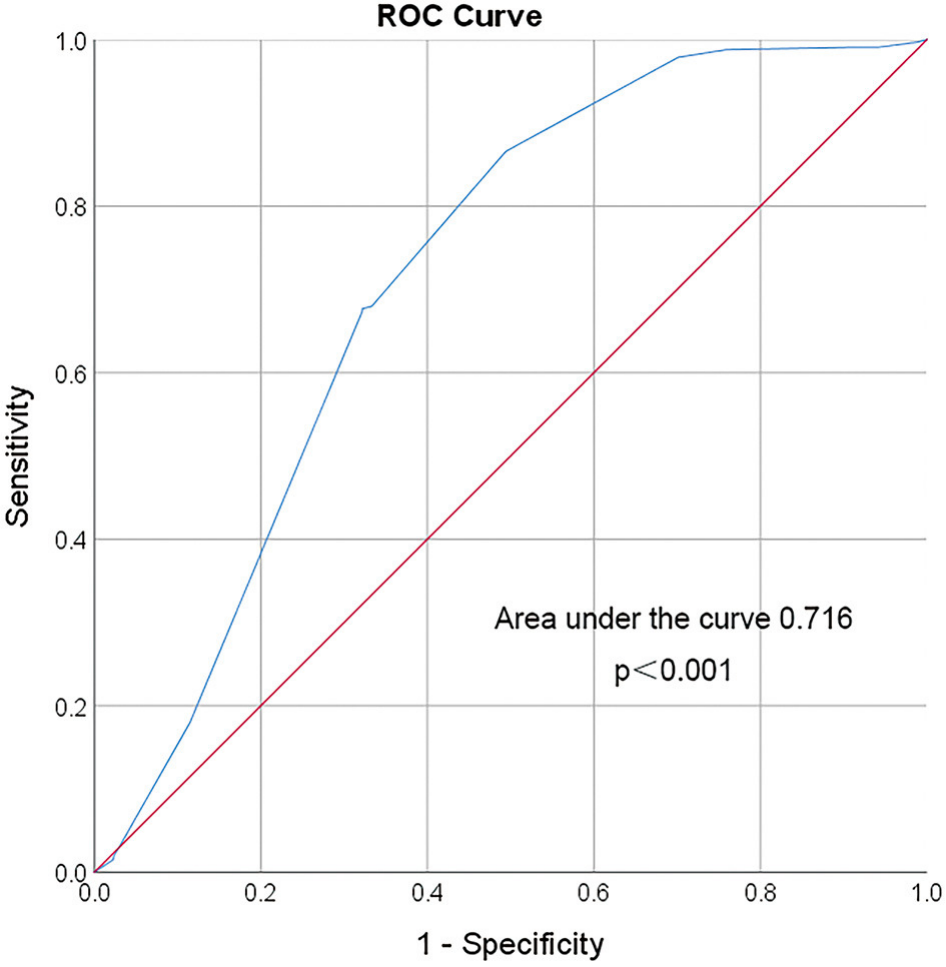
ROC curve of the lowest SBP before VA ECMO initiation. Area under the ROC Curve of the lowest systolic blood pressure (72.5 mmHg) before VA ECMO initiation for predicting incidence of neurologic complications during ECMO is 0.716 (95% CI 0.647–0.786). The red line indicates reference values.

In converting the lowest SBP level before V-A ECMO initiation from a continuous to a categorical variable, 72.5 mmHg was chosen as a threshold. It offered a sensitivity and specificity of 86.6 and 50.6% for prediction, respectively. The odds ratio of neurologic complications associated with SBP lower than 72.5 mmHg was 6.61 (95% CI: 3.90–11.19, *p* < 0.001; Figure 6).

## Discussion

This study shows the neurologic complications in adult PCS patients receiving V-A ECMO support. We found that the rate of successful weaning from V-A ECMO and survival to discharge were significantly lower in patients with neurologic complications. The lowest SBP level pre-ECMO and aortic surgery combined with CABG were identified as correlative factors independently associated with overall neurologic complications in these patients.

In this study, neurologic complications occurred in 21% of cases in adult PCS patients supported with V-A ECMO, a slightly higher rate reported by previous investigations (6–17%) (12, 15, 21, 22). This perhaps was due to the type of cardiac surgery or by neurologic patient examination protocols. In previous studies, owing to the difficulties of getting an imaging examination during V-A ECMO support or patients dying from severe neurologic complications without an imaging examination, the true incidence of neurologic complications might have been underestimated (14–16, 18, 21–25).

Matteen et al. (23) found that increased age was associated with higher rates of death and neurological morbidity. We also found that patients with neurologic complications were older, obese, and with more comorbidities, especially hypertension, hyperlipidemia, and peripheral arterial disease. All of these conditions indicate patients' worse general status and vascular condition in our series, which may lead to increased rate of neurologic complications.

We found that the incidence of neurologic complications was associated to pre-ECMO low perfusion situations. ECPR was also frequently present in patients with neurologic complications. The incidence of neurologic complications in ECPR patients was higher than that in the patients failure to wean off CPB or the patients of postoperative LCOS. The reasons may be that the patients failing to wean off CPB (or postoperative LCOS patients) could promptly transition to V-A ECMO and whereas the majority of ECPR patients may have experienced cerebral hypo-perfusion, hypoxia, and reperfusion injury prior to ECMO implantation (23). Moreover, aortic surgery combined with CABG was shown to be an independent risk factor of the neurologic complications in the current study. The possible reason could be that the patients in our study had type A aortic dissection involving coronary artery and multiple organs, requiring intraoperative deep hypothermic circulatory arrest and long CPB times, conditions more likely associated with postoperative neurological injury.

Coagulation disorders can be induced by many factors, including the ECMO circuit, the surgical procedures, and the severity of the disease. Coagulation disorders were important factors affecting the integrity of the neurologic system and might lead to intracranial hemorrhage (12). In addition, we found that a history of atrial fibrillation was an independent risk factor of both intracranial hemorrhage and cerebral infarction. Atrial fibrillation is a well-known reason for thromboembolism in PCS patients (26). On the other hand, the patients with atrial fibrillation need anticoagulants to prevent thrombosis, condition favoring the occurrence of intracranial hemorrhage. Our results are in accordance with the previous literature regarding intracranial bleeding occurring in ECMO patients (27–29). Hyperlipidemia was associated with seizures which may be due to asymptomatic cerebrovascular disease secondary to dyslipidemia, but the underlying mechanism is yet less defined and warrants further research. A threshold of lowest SBP before V-A ECMO initiation, which may predict prognosis and assist doctors in managing patients, was defined in our investigation. When patients' blood pressure cannot be sustained by vasopressors, ECMO should be used prior to severe and refractory hypotension. In the clinical setting, neurologic complications may be induced by multiple factors during V-A ECMO support. Similarly, risk factor identification may help initiate steps to lower the risk of such complications in PCS patients undergoing temporary ECMO assistance.

### Limitations

This single-center study is limited by its retrospective nature. The exact timing of neurologic complication in relation to ECMO and information regarding neurologic impairment before ECMO were uncertainty. In addition, full neuroimaging assessment was not performed on every patient. Even though we could perform CT scan, we had no MRI results because of the restrictions of the ECMO device, and, therefore, some subtle abnormalities, such as cerebral microbleeds, might have been not objectivated (30). Therefore, neurological complications were likely underestimated. However, routine neurological examinations were performed at least twice a day by the ICU staffs. It was unlikely to miss neurological complications with positive clinical manifestations in this study. Neurological events after ECMO weaning were not involved in this study, because of confounding factors. Another limitation is that we did not have long-term follow-up on survivors.

## Conclusions

Neurologic complications are frequent in adult PCS patients treated with VA ECMO, and are associated with increased in-hospital mortality. We identified the lowest SBP level before V-A ECMO initiation, CABG combined with aortic surgery, age, coagulation disorders, atrial fibrillation and hyperlipidemia as independent risk factors for different neurologic complications during V-A ECMO support in PCS patients.

## Data Availability Statement

The raw data supporting the conclusions of this article will be made available by the authors, without undue reservation.

## Ethics Statement

This study was approved by Beijing Anzhen Hospital human research Ethics Committee (Ethics number: 2016018X). Because this was a retrospective observational study, the individual patients' consent was waived.

## Author Contributions

DH and HW participated in the design of the study, analyzed the data, and drafted the manuscript. FY interpreted the data and revised the manuscript. XH conceived the study, participated in its design, and revised the manuscript. All authors contributed to the article and approved the submitted version.

## Conflict of Interest

The authors declare that the research was conducted in the absence of any commercial or financial relationships that could be construed as a potential conflict of interest.

## Publisher's Note

All claims expressed in this article are solely those of the authors and do not necessarily represent those of their affiliated organizations, or those of the publisher, the editors and the reviewers. Any product that may be evaluated in this article, or claim that may be made by its manufacturer, is not guaranteed or endorsed by the publisher.

## Abbreviations

ACT: activated clotting time
aPTT: activated partial thromboplastin time
BMI: body mass index
CABG: coronary artery bypass grafting
CI: confidence interval
CNS: central nervous system
COPD: chronic obstructive pulmonary disease
CPB: cardiopulmonary bypass
CPR: cardiopulmonary resuscitation
CRRT: continuous renal replacement therapy
CT: computed tomography
ECMO: extracorporeal membrane oxygenation
ECPR: extracorporeal cardiopulmonary resuscitation
FFP: fresh frozen plasma
IABP: intra-aortic balloon pump
ICU: intensive care unit
IQR: interquartile range
LCOS: low cardiac output syndrome
MV: mechanical ventilation
OR: odds ratio
PCS: post-cardiotomy cardiogenic shock
RBC: red blood cells
SBP: systolic blood pressure
VA ECMO: veno-arterial extracorporeal membrane oxygenation.
